# A Theoretical Study on Trehalose + Water Mixtures for Dry Preservation Purposes

**DOI:** 10.3390/molecules25061435

**Published:** 2020-03-21

**Authors:** Amit Kumar, Alberto Cincotti, Santiago Aparicio

**Affiliations:** 1 Department of Electrical and Electronic Engineering, University of Cagliari, 09123 Cagliari, Italy; amit.kumar@unica.it; 2 Department of Mechanical, Chemical and Materials Engineering, University of Cagliari, 09123 Cagliari, Italy; alberto.cincotti@dimcm.unica.it; 3 Department of Chemistry, University of Burgos, 09001 Burgos, Spain

**Keywords:** trehalose, water solutions, dry preservation, hydrogen bonding molecular dynamics

## Abstract

The properties of trehalose + water mixtures are studied as a function of mixture composition and temperature using molecular dynamics simulations. As trehalose disaccharide has been proposed for dry preservation purposes, the objective of this work is to analyse the nanoscopic properties of the considered mixtures, in terms of aggregation, clustering, interactions energies, and local dynamics, and their relationships with hydrogen bonding. The reported results allow a detailed characterization of hydrogen bonding and its evolution with mixture composition and thus inferring the effects of trehalose on water structuring providing results to justify the mechanisms of trehalose acting as preservation agent.

## 1. Introduction

**Figure 1:**
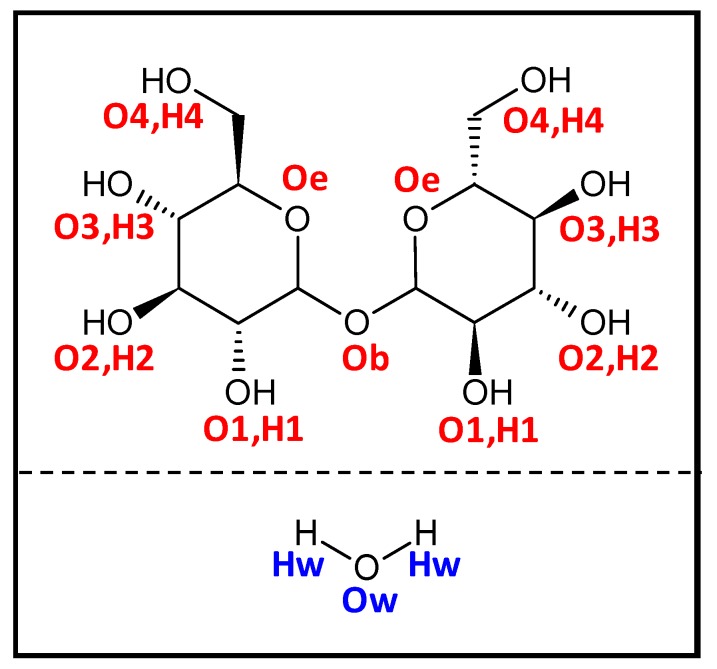
Structure of Trehalose (THAL) and water molecules with atom labelling used in this work.

Trehalose (THAL, Figure 1), a non-reducing disaccharide, is composed of two glucose units that are linked together with an α, α-1,1-glycosidic linkage, with interesting properties that have found application in the food, cosmetics and pharmaceutical industry [1]. In the year 2002, US Food and Drug Administration (FDA) approved trehalose as a safe food ingredient [2]. The wide range of applications of this sugar has increased the interest of many researchers towards development of novel and economically feasible production systems [3].

One of the most commonly practises for preservation and storage of biological samples is freeze-drying [4]. However, some undesirable side effects, such as denaturation of sensitive proteins and decreased viability for many cell types can damage to biological systems resulting from freeze drying. According to the water replacement hypothesis, THAL was found to play a key role in the survival of plants and insects, in harsh environments, even when most of their water body is removed, due to its exceptional capability of hydrogen bonding with biological macromolecules [5]. Thus, the portrayal of properties of these types of organisms drove attention towards investigation of THAL. It has been shown that the presence of THAL in biological systems such as lipids, proteins and tissues, confers improved tolerance against freezing and desiccation, wherein THAL readily dries as an amorphous material with a high glass transition temperature (*T_g_* > 100 °C). Therefore, this feature of THAL has been exploited in cryopreservation of biological matter [6]. It has been shown that addition of water causes decrease in the *T_g_* for THAL due to plasticizing effect; however, with respect to other disaccharide at similar water content, THAL displays higher value of *T_g_* [7]. Considering these physicochemical advantages, THAL has been explored as a nontoxic protective additive to preserve biospecimen including stem cells and tissue-engineered skin [8].

Glass transition is associated to phenomena observed when a glass is heated until it behaves like a super cooled melt, which can be measured experimentally using differential scanning calorimetry (DSC) technique [9]. There seems to be a strong correlation between the glass transition temperature of biomaterials and its stability. Prior knowledge of glass transition temperature allows controlling the rate of physical, chemical and biological changes.

Computational methodology, such as molecular dynamics (MD) simulations of pure and aqueous THAL systems, have been carried out previously to probe the thermodynamic properties associated to glass transition [10]. These modelling results for dehydration processes probed the formation of small THAL clusters for water contents larger than 0.1 g water/gDW whereas for concentrations lower than 0.1 g water/gDW giant THAL clusters are formed. These results agree with the experimental results showing that the protective effect of THAL on cells viability decreases when anhydrobiosis approached the 0.1 g water/gDW level [11] and thus confirming the existence of a critical water content for dry preservation purposes related with the changes in aggregation and clustering in water-THAL mixtures and with the evolution of hydrogen bonding with water content and temperature.

The objective of this work is to advance in the understanding of intermolecular interactions (hydrogen bonding) and clustering in THAL-water mixtures by using molecular dynamics (MD) simulations considering the whole composition range (Table S1, Supplementary Material) and analysing the 100 to 400 K temperature range. Physicochemical properties amongst THAL molecules and between THAL and water molecules were quantitatively investigated, in particular, investigation of energetics, hydrogen bonding, radial distribution (clustering) and diffusion properties at different concentrations and temperatures.

## 2. Results and Discussion

DFT Results. The properties of THAL + water mixtures stand on the hydrogen bonding abilities of both types of molecules, which should lead to an extensive network of interactions along the liquid structure. Therefore, the characteristics of these mixed fluids are produced by the nature of the possible THAL-water interactions, which can be accurately analysed by using DFT methods. For this purpose, clusters with 1 THAL + *i* water (*i* = 1 to 3) were optimized with DFT approach, thus providing information of water clustering around THAL molecules. Although interactions in liquid state will extend beyond these simple cluster models, the characterization provided by DFT calculations leads to information which can be extended through bulk liquid phased by MD simulations.

**Figure 2:**
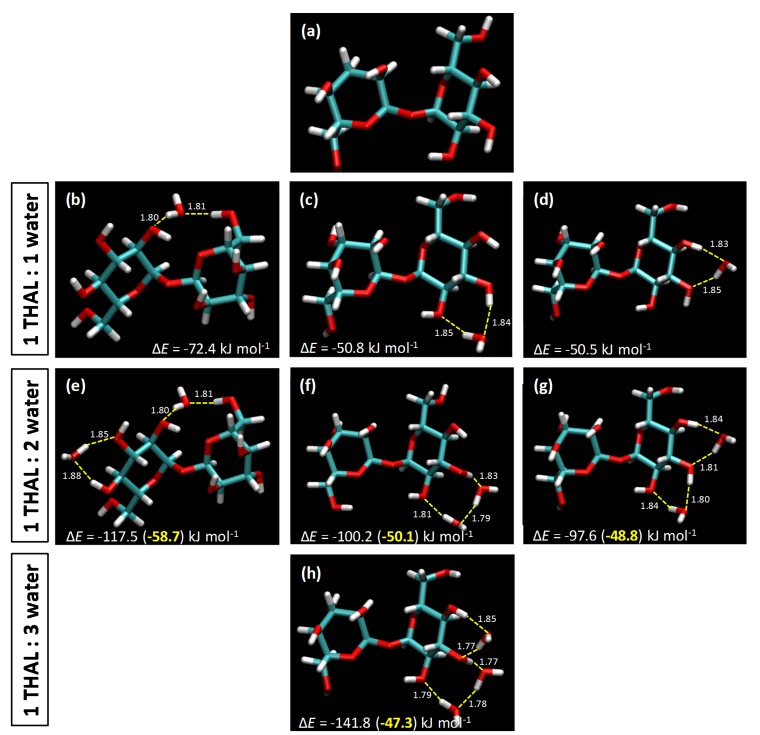
Structure of (**a**) THAL and (**b**–**h**) 1 THAL + *i* water clusters (*i* = 1 to 3) optimized from DFT calculations. Dashed yellow lines show relevant THAL-water hydrogen bonds, with interaction distance in Å. Interaction energies, Δ*E*, are also reported.

In a first stage, the structure of THAL monomers was optimized at the considered theoretical level (B3LYP-D3/6-311++g(d,p)), Figure 2a. The α,α isomer reported in Figure 2a is the naturally occurring form of THAL [12], as well as the lowest energy conformer [13], and thus, it was the only isomer considered for DFT calculations. Regarding the THAL structure, the presence of four hydroxyl groups in each hexose ring, which are fully exposed to external solvent molecules being not sterically hindered in contrast with the glycoside oxygen, which would be hindered for accepting hydrogen bonds, should lead to extensive interactions with surrounding water molecules, i.e., water–THAL hydrogen bonding can be developed through any of the eight hydroxyl groups with similar topological characteristics and strengths.

For the case of 1 THAL:1 water clusters (Figure 2b,d), the most relevant interaction sites were considered: (i) water acting as a bridge between the two hexoses involving the hexose -CH2-OH group (Figure 2b) and (ii) water bridging two hydroxyl groups in one of the hexoses at two different sites (Figure 2c,d). The case of water bridging the two hexoses leads to larger binding energies than in the case of water molecules hydrogen bonding a single hexose ring (roughly 42% stronger interactions), with the water molecule developing hydrogen bonds with hydroxyl groups at roughly the same distances, thus pointing to strong hydrogen bonds with both rings; Figure 2b. Likewise, the connection of the two hexose rings in Figure 2b leads to large changes in THAL molecule, with changes in the relative disposition of the two hexose rings around the glycoside oxygen. The structure of THAL bonded to water in Figure 2b is more compact than the one for isolated THAL in Figure 2a, the calculated Connolly volumes being 295.24 and 301.04 Å^3^, respectively. The development of water-THAL hydrogen bonding in a single hexose ring (Figure 2c,d) leads to large binding energies but lower than those in Figure 2b. In any case, water molecules develop two simultaneous hydrogen bonds with hydroxyl groups in hexose rings with similar H to O distances pointing in all the cases to very effective hydrogen bonding.

**Figure 3:**
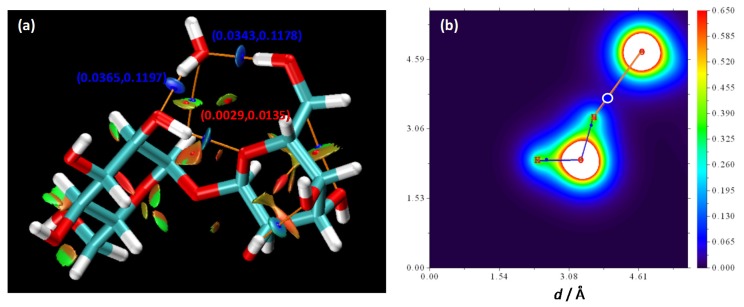
(**a**) AIM and RDG analysis of 1:1 THAL:water cluster. AIM binary (3, −1) and ring (3, +1) critical points are showed as blue and red spheres, respectively. AIM bond paths are showed as orange lines. For relevant critical points (electron density and Laplacian of electron density), a.u. are reported. Colour code for RDF isosurfaces: (blue) hydrogen bonding, (green) van der Waals interactions and (red) repulsive interactions. (**b**) Contour map of electron density for the region of water–THAL interaction (water acting as hydrogen bond donor).

Results in Figure 3 show AIM and RDG (Reduced Density Gradient) analysis for the 1:1 interacting pair in Figure 2b. This interaction is characterized by the development of two binary critical points (BCPs, type (3, −1)) along the donor–acceptor line, closer to the corresponding H atoms, for which the electron density, $\rho_{e}$, and the corresponding Laplacian, $\nabla^{2}\rho_{e}$, are in the upper limit of the corresponding ranges considered for defining hydrogen bonding in the AIM framework (0.002 to 0.035 a.u., 0.024 to 0.139 a.u, for $\rho_{e}$ and $\nabla^{2}\rho_{e}$, respectively) [14]. Therefore, although some doubts have been raised in the literature about the quantification of hydrogen bonding by AIM parameters [15], the results for BCPs point to very strong hydrogen bonds according to interaction energies in Figure 2. Likewise, the interacting water-THAL region is characterized by the development of two ring critical critical points (RCPs, type (3, +1)), which confirm the reinforcement (cooperativity) of the binding by the presence of the two simultaneous interactions connecting both hexose rings [16]. The analysis of the hydrogen bonding in terms of the Noncovalent interaction (NCI) analysis [17] showing the Reduced Density Gradient (RDG) surfaces is also reported in Figure 3a. The two large blue spots in the water -THAL hydrogen bonding bond path show strong attraction corresponding to the large $\rho_{e}$ and negative λ_2_ values (being λ_2_ the largest eigenvalue of Hessian matrix of electron density). Additionally, the green spot around the RCPs confirms the reinforcement of the interaction by additional van der Waals interaction. The contour map of electron density in one of the water-THAL hydrogen bonds in Figure 3b shows the densification along the hydrogen bonding bond path. For the case of 1 THAL:2 water and 1 THAL:3 water clusters (Figure 2e–h), the reported results show that water molecules can be hydrogen bonded to neighbour sites in THAL molecule without weakening the THAL-water strength of interaction, the trend of energetics being maintained, thus leading to a cooperativity effect, which would lead to efficient THAL solvation in liquid water solutions.

MD simulations. The DFT results concluded strong hydrogen bonding between water and THAL molecules, which would compete in the liquid state with the strong term of THAL molecules to self-aggregate to form large clusters [10]. These effects were analysed in this work by MD simulations as a function of mixture composition and temperature considering large ranges to study different dry preservation scenarios.

**Figure 4:**
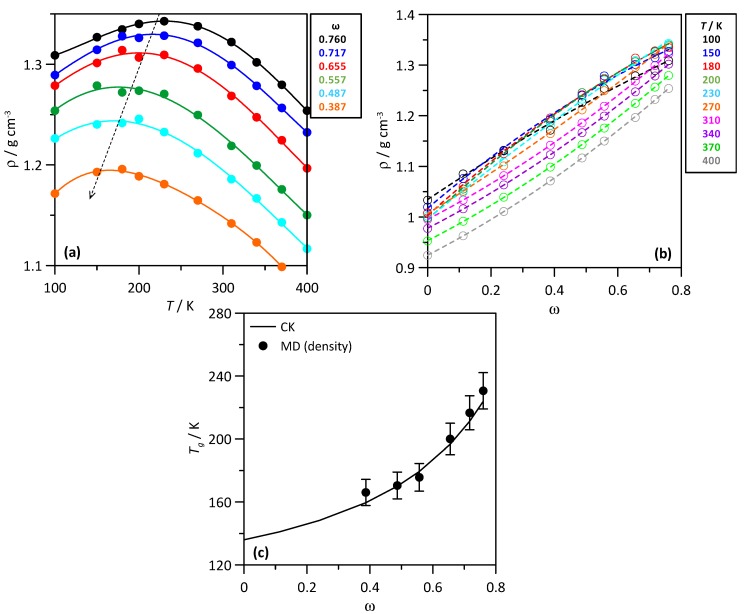
(**a**,**b**) Density, ρ, and (**c**) glass transition temperature, *T_g_*, for ω THAL + (1-ω) water mixtures. *T* stands for temperature and ω for THAL mass fraction. Values in panel c were obtained from the maxima of density vs. *T* curves (MD) and compared with those predicted from CK model. In panel a, dashed arrow indicates the shifting of curves maxima with decreasing THAL content.

Water-THAL mixtures are characterized by the formation of glassy states characterized by the corresponding glass transition temperatures, *T_g_* [18]. The formation of glassy structures was inferred from MD simulations by the mixture composition evolution of predicted density (Figure 4a), which evolve through maxima with temperature for each mixture, thus allowing the prediction of *T_g_* (Figure 4c). Predicted *T_g_* were compared with those from Couchman–Karasz (CK) model, which showed excellent agreement with experimental results [19]. The MD predicted *T_g_* are in excellent agreement with those from CK model. The maxima of density vs. *T* curves shifts towards lower temperatures as the water content increases. A non-linear evolution of *T_g_* vs. THAL mass fraction (ω) results, which is in agreement with the literature data [10] showing the trend of THAL molecules to self-aggregate forming giant THAL clusters as the water content decreases (increasing ω in Figure 4c), whereas the large *T_g_* point to small THAL clusters for water rich mixtures. The evolution of predicted MD density with mixture composition for isothermal conditions as reported in Figure 4b also shows non-linear evolution, especially for low temperatures, which agrees with the clustering and thus glassy states for THAL rich mixtures.

**Figure 5:**
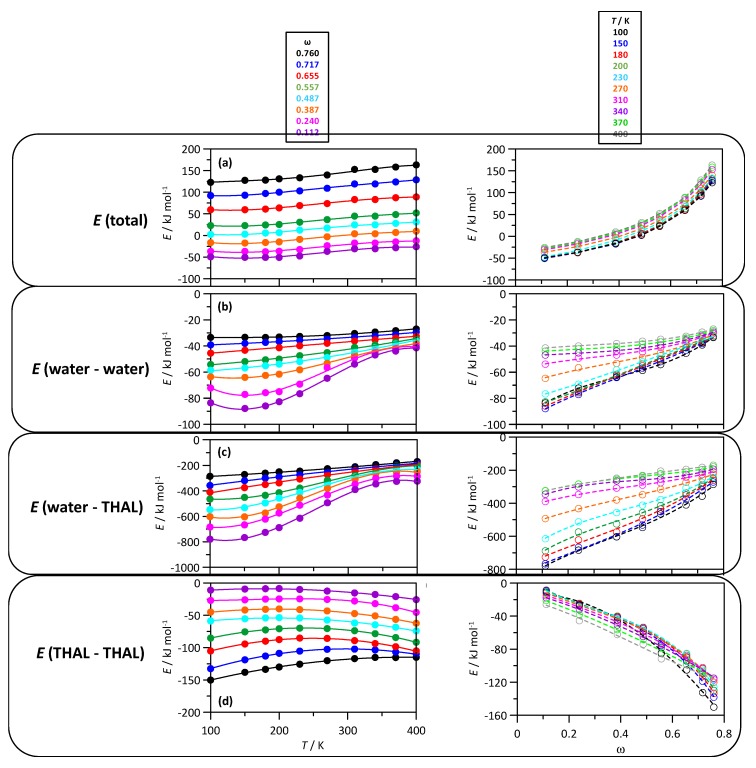
Evolution of relevant energy terms with mixture composition and temperature for ω THAL + (1-ω) water mixtures. *T* stands for temperature and ω for THAL mass fraction. *E*(total) stands for the total potential energy; *E*(water-water), *E*(water-THAL) and *E*(THAL-THAL) for the intermolecular interaction energies (sum of Lennard–Jones and coulombic contributions) of the corresponding interacting pairs.

The strength of intermolecular forces from MD simulations are reported in Figure 5. The total potential energy, *E*(total), is reported in Figure 5a as a function of temperature and composition. The *E*(total) of the system provides an estimate about the stability of the systems under investigation. In general, we observe the stability of the molecular system decreases upon increasing the concentration of THAL and upon increasing the temperature. Mixtures with low THAL content show negative *E*(total) in the full temperature range, whereas as the THAL content increases, positive *E*(total) are inferred, which can be related with phase segregation through THAL large clustering. Additionally, the composition evolution of *E*(total) vs. THAL content under isothermal conditions is largely non-ideal for all the considered temperatures, which points to large changes in the aggregation schemes, i.e., type and size of homo and heteroassociated clusters, with composition evolution. The interaction energy was split in the different contributions: water-water, water-THAL and THAL-THAL; Figure 5b–d. The water-THAL interactions (heteroassociations) lead to larger interaction energies than the developed homoassociations (water-water and THAL-THAL). The homoassociations are stronger for the corresponding mixture rich regions, i.e., water–water for water rich mixtures and THAL-THAL for THAL rich mixtures. It is especially remarkable for THAL-THAL interactions, which evolve non-linearly with mixture composition reaching very large values, equivalent to water-THAL ones, for THAL rich mixtures. This behaviour agrees with the increasing trend of THAL molecules to self-associate reaching large clusters for THAL rich mixtures, i.e., leading to very large THAL-THAL interaction energy with the decrease of water-THAL interactions.

**Figure 6:**
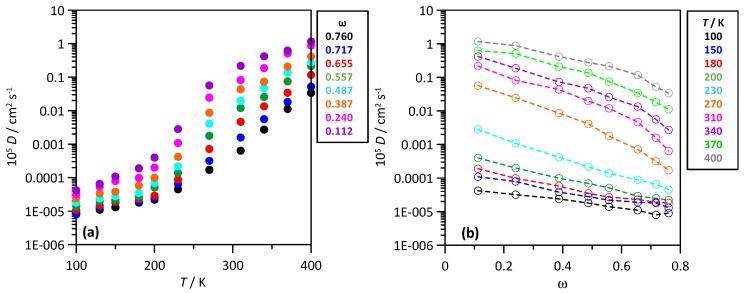
Self-diffusion coefficients of THAL, *D*, in ω THAL + (1-ω) water mixtures. *T* stands for temperature and ω for THAL mass fraction.

The development of strong hydrogen bonding in the mixtures as well as its non-linear evolution with mixture composition should lead to different molecular mobility in the mixtures considering THAL content and temperature. This effect was analysed through the MD predicted self-diffusion coefficients, *D*, which were calculated from mean square displacements and Einstein’s equation for fully diffusive regimes. The temperature evolution of THAL *D* values reported in Figure 6a shows a non-linear evolution corresponding to the formation of a glassy state for the corresponding *T_g_* for each mixture composition. The large deviations of Arrhenius behaviour for THAL self-diffusion as reported in Figure 6a manifest cooperativity of molecular diffusion. The cooperativity is maintained in the whole concentration range, thus suggesting THAL trend to clustering. Likewise, the decrease of THAL *D* values with increasing THAL content confirms the THAL aggregation.

**Figure 7:**
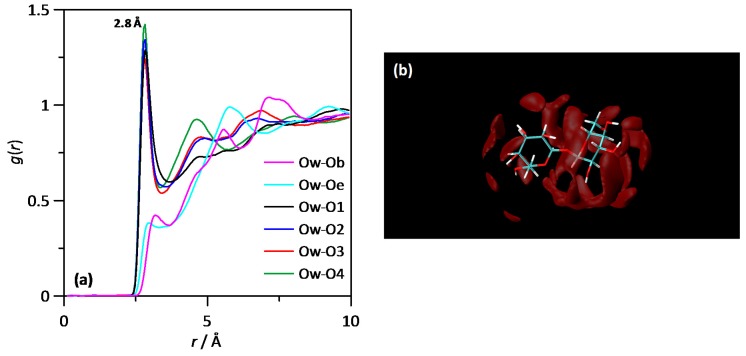
(**a**) Site-site radial distribution functions, *g*(*r*), for the reported atomic pairs; (**b**) spatial distribution functions of oxygen atoms in water (Ow) around THAL molecule. All the results reported for ω THAL + (1-ω) water mixture (ω = 0.240) at 310 K. Atom labelling as in Figure 1.

The structuring of water–THAL mixtures is firstly analysed by site-site radials distribution functions, RDFs. The hydrogen bonding between water and THAL molecules considering the different donor/acceptor sites in THAL is analysed in Figure 7a. The reported results discard the formation of hydrogen bonds through the oxygen linking the two hexose rings, Ob, and through the two ether oxygen atoms in the hexose rings, Oe (atom labelling in Figure 1); Figure 7a. Regarding the interaction with the four possible hydroxyl groups in each ring, O1 to O4 (Figure 1), the corresponding oxygen (water) to oxygen (OH in THAL) RDFs are characterized by a strong and narrow peaks at 2.8 Å, showing strong hydrogen bonds in agreement with DFT results in Figure 2. The intensity of these RDFs first peaks is almost the same for all the available hydroxyl THAL groups, thus confirming that water molecules are hydrogen bonded to all the available sites although with slightly different extension around each site. The differences arise in the water structuring beyond the first solvation shell (hydrogen bonds); the second water solvation shell is inferred from the second RDF peak, whose intensity increases from O1 to 4 OH groups, i.e., larger RDFs as the OH groups are further from the bridge oxygen.

**Figure 8:**
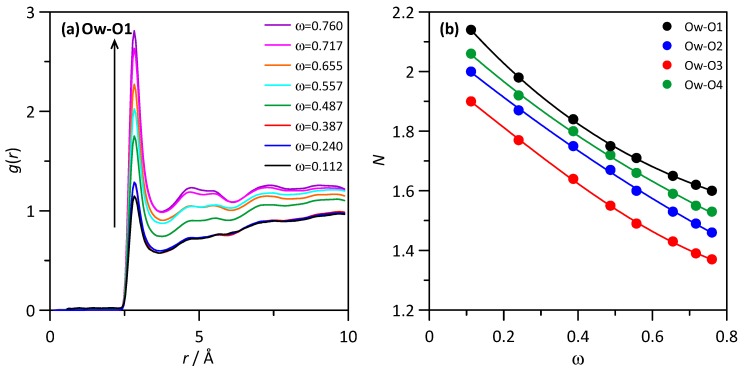
(**a**) Site-site radial distribution functions, *g*(*r*), and (**b**) running integrals, *N*, for the reported atomic pairs in ω THAL + (1-ω) water mixture at 310 K. The arrow indicates increasing ω. Atom labelling as in Figure 1.

**Figure 9:**
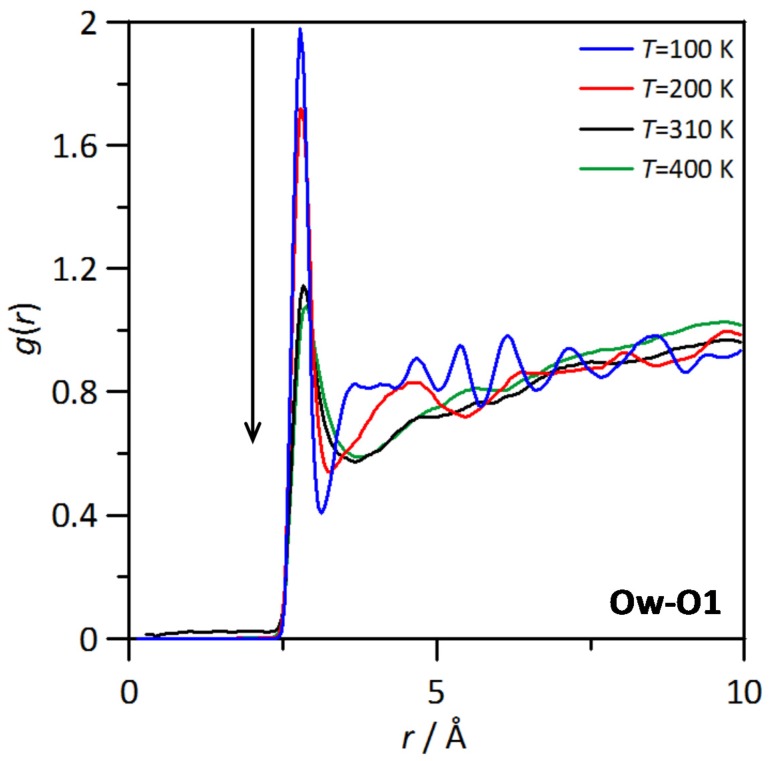
Site-site radial distribution functions, *g*(*r*), for the reported atomic pairs in ω THAL + (1-ω) water mixture (ω = 0.112) as a function of temperature. Atom labelling as in Figure 1.

**Figure 10:**
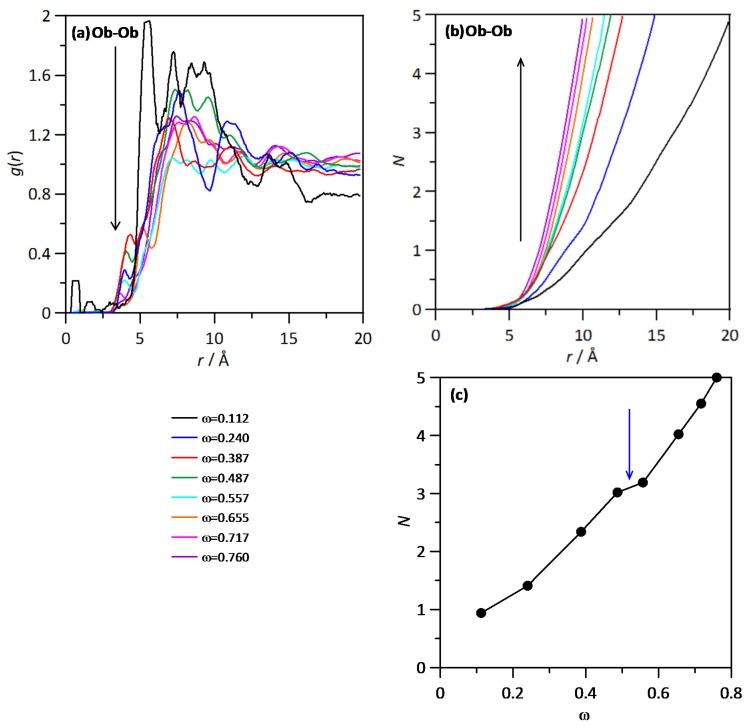
(**a**) Site-site radial distribution functions, *g*(*r*), and (**b**) running integrals, *N*, for the reported atomic pairs in ω THAL + (1-ω) water mixture at 310 K. The arrows indicate increasing ω. Atom labelling as in Figure 1. Results in panel (**c**) show *N* for *r* < 10 Å as a function of mixture composition.

The spatial distribution function of water molecules around a central THAL molecule is reported in Figure 7b, which shows water molecules concentrating around the two hexose rings, although the distribution is not homogeneous with larger water concentration around one of the hexose ring in comparison with the other one, which can be justified considering that most of the water molecules will be placed in the outer cores of THAL-THAL clusters, thus with a part of the THAL molecule involved in THAL-THAL interactions and the other part (second hexose ring) exposed to water and thus leading to water-THAL hydrogen bonding in larger extension. The composition effect on RDFs is analysed (at 310 K) in Figure 8a, where Ow (water)–O1 (THAL) as a function of THAL content; the reported results show that RDF first peaks is maintained in the whole composition range with very minor changes in RDFs beyond the peak corresponding to hydrogen bonding. The integration of RDFs leads to the corresponding running integrals, *N*, i.e., the number of water molecules around each THAL hydroxyl site; Figure 8b. The *N* values in Figure 8b follows the ordering O1 > O4 > O2 > O4 > O3, i.e., larger concentration of water molecules directly hydrogen bonded to THAL for OH groups closer to the O bridge site. Nevertheless, the differences are minor, and for low THAL content, *N* values are close to two for all the considered sites, thus confirming that water molecules are hydrogen bonded to O1–O4 sites. The increasing THAL concentration leads to a decrease of *N* following a non-linear evolution with THAL mass fraction. This behaviour can be justified with the increase of THAL-THAL clusters, which decrease the free available OH sites to be hydrogen bonded to water molecules. The non-linear evolution of *N* shows that THAL clusters do not increase their size just because the increase of THAL content, with the size increasing abruptly from a certain THAL content [10]. The temperature effect on water–THAL aggregation is reported in Figure 9 in the 100 to 400 K range (for ω = 0.112). The reported results show that although the position of the first RDF peak is maintained in the whole temperature range, i.e., water and THAL are hydrogen bonded in the whole range; the intensity of this first peak decreases upon heating with a large change through the *T_g_* and large differences in the ordination beyond the first solvation shell, which correspond to different structural arrangements in the glassy and liquid states. Regarding the THAL-THAL interactions, RDFs for Ob-Ob (as centre-of-mass to centre-of-mass interactions) are reported in Figure 10a for isothermal conditions (*T* = 310 K) as a function of THAL content. The reported RDFs show peaks in the 5 to 10 Å range, with narrower peaks for water rich mixtures evolving to wider bands as THAL concentration increases. Therefore, it shows THAL clustering, which is confirmed by the corresponding *N*s in Figure 10b, the non-linear increase of THAL molecules around a central THAL shows increase of THAL-THAL clusters sizes but with an abrupt increase beyond certain water THAL content as showed in Figure 10c.

**Figure 11:**
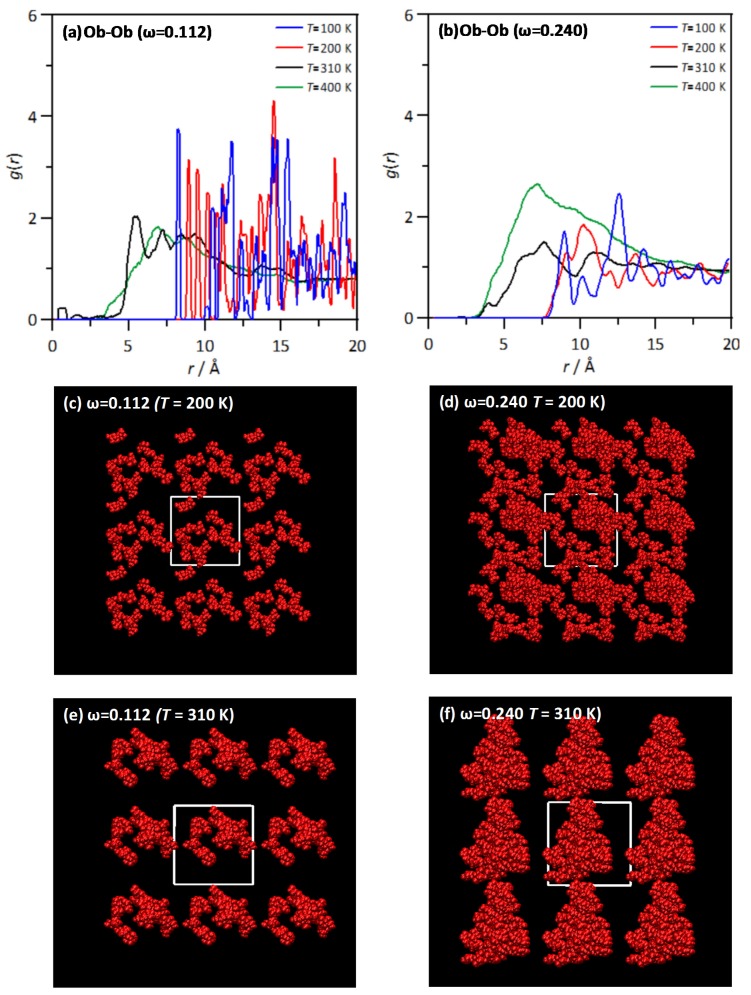
(**a**,**b**) Site-site radial distribution functions, *g*(*r*), for the reported atomic pairs in ω THAL + (1-ω) water mixtures as a function of temperature. (**c**–**f**) Snapshots, after 20 ns simulations, of distribution of THAL molecules at selected temperatures for the reported mixtures’ composition, with water molecules omitted for the sake of visibility. In panels (**c**–**f**) white lines indicate periodic boundary conditions, THAL molecules are replicated outside the simulated region (white lines) for showing periodicity of THAL aggregation. Atom labelling as in Figure 1.

The THAL clustering is analysed as visualized in Figure 11. RDFs for Ob-Ob sites (THAL-THAL self-association) are plotted for two THAL contents as a function of temperature as well as the corresponding snapshots showing THAL clusters. For low THAL content (Figure 11a) RDFs show poor ordering for low temperatures and thus only once the *T*_g_ is surpassed, the clustering is inferred. Analogous results are inferred as the THAL content is increased, although the RDFs show wider bands corresponding to larger THAL-THAL aggregates; Figure 11b. The snapshots reported show poorly defined THAL clusters for low temperatures below the *T*_g_ whereas clusters are properly defined once *T*_g_ is surpassed. The increase in cluster size with increasing THAL content is clearly inferred from results in Figure 11e,f; the massive clusters (even propagating through neighbour simulated cells) at ω = 0.240 contrast with the smaller clusters for ω = 0.112, which confirm that THAL-THAL homoassociations are very favoured, and massive [10] clusters are formed thus leading to water molecules solvating, by hydrogen bonding, the external shells of these large clusters.

**Figure 12:**
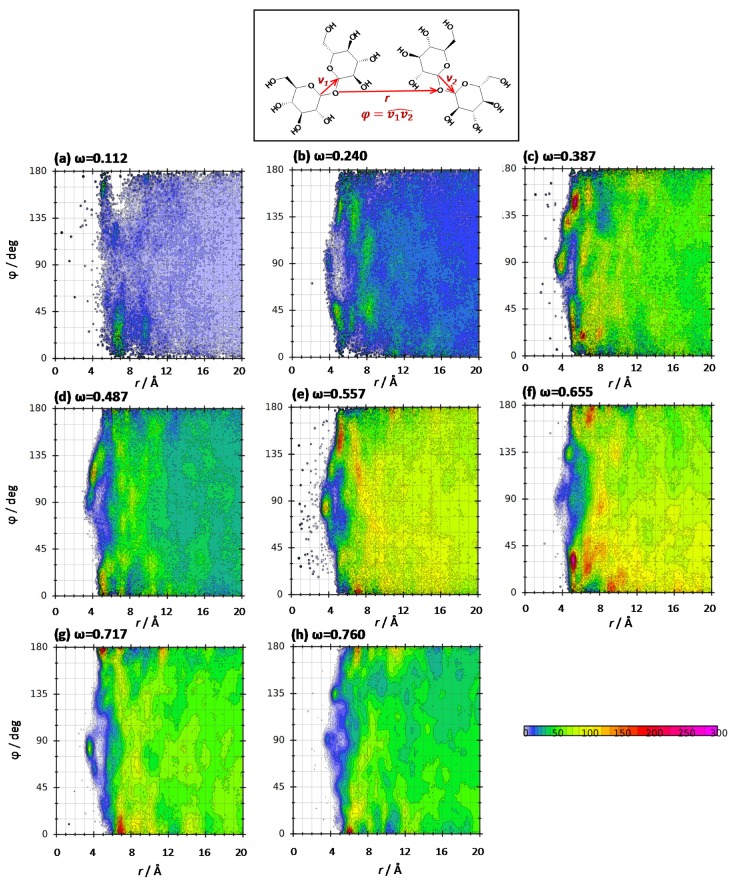
Combined distribution function for the reported distance and angle in ω THAL + (1-ω) water mixtures at 310 K.

**Figure 13:**
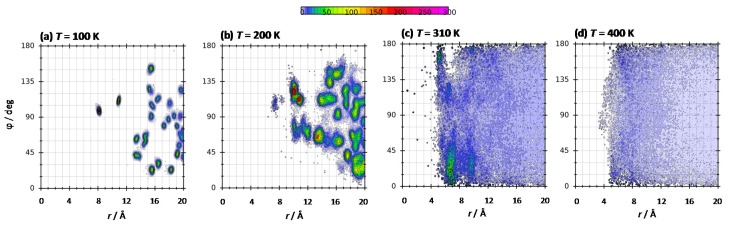
Combined distribution function for the reported distance and angle in ω THAL + (1-ω) water mixtures (ω = 0.112) as a function of temperature. Angle, ϕ, and distance, *r*, defined as in Figure 12.

The orientation of THAL molecules in THAL-THAL large clusters was quantified by combined distribution functions, CDFs (plotted using TRAVIS software [20]), for the vectors joining two opposite carbon atoms in the hexose rings and the Ob-Ob intermolecular distance. It should be remarked that, as results in Figure 7b show, the two hexose rings are not coplanar, which agrees with DFT results in Figure 2. Results in Figure 12 show a strong composition dependence on CDFs, as it may be expected considering the different geometries of THAL clusters reported in Figure 11e,f. As a rule, for most of the mixture compositions no large spots for the studied angle are inferred, thus discarding parallel orientation of neighbour THAL molecules. Therefore, stacking is not inferred and THAL molecules seem to be mostly randomly oriented for leading to the clusters reported in Figure 11e. The temperature effect on CDFs is reported in Figure 13 showing the poor THAL clustering below the *T_g_*, in agreement with the results in Figure 11 for cluster properties. Additionally, large temperatures (e.g., 400 K in Figure 13d) lead to less ordered THAL clusters.

**Figure 14:**
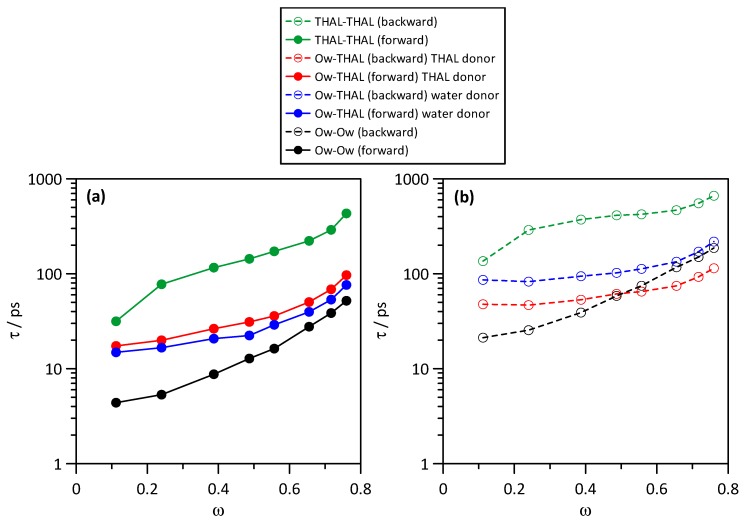
Kinetic parameters of hydrogen bonding for the reported interacting sites showing relaxation times, τ, for the (**a**) forward, i.e., hydrogen bond lifetime, and (**b**) backward, i.e., hydrogen bonding reformation after destruction, processes in ω THAL + (1-ω) water mixtures at 310 K. Atom labels as in Figure 1. For THAL molecule, all available OH sites (O1 to O4, Figure 1) are considered as donor or acceptors.

**Figure 15:**
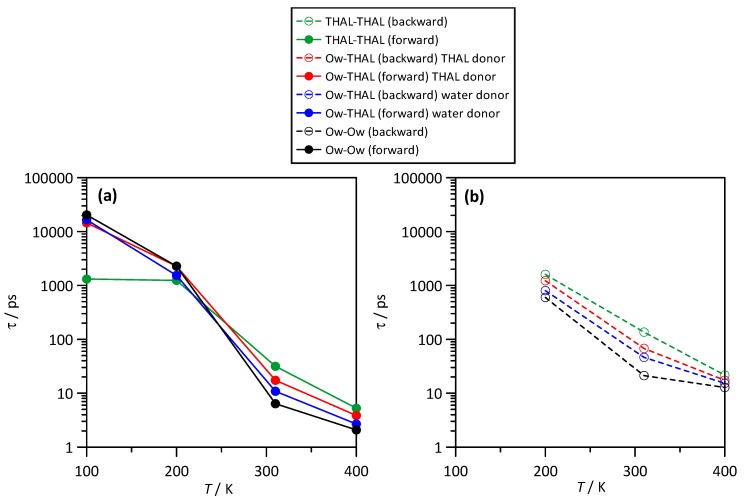
Kinetic parameters of hydrogen bonding for the reported interacting sites showing relaxation times, τ, for the (**a**) forward, i.e., hydrogen bond lifetime, and (**b**) backward, i.e., hydrogen bonding reformation after destruction, processes in ω THAL + (1-ω) water mixtures (ω = 0.112) as a function of temperature. Atom labels as in Figure 1. For THAL molecule, all available OH sites (O1 to O4, Figure 1) are considered as donor or acceptors.

The kinetic properties of the different hydrogen bonds were analysed by using the reactive flux method by Gehrke et al. [21] using the forward (hydrogen bond dissociation) and backward (hydrogen bond reformation) processes characterized by the corresponding lifetimes, $\tau_{forward}$ and $\tau_{backward}$. Therefore, the $\tau_{forward}$ can be considered to quantify the duration of the corresponding hydrogen bonds as well $\tau_{backward}$ to measure the difficulties to form a new hydrogen bond after breaking. Hydrogen bonds were defined with 3.5 Å and 60° as criteria for donor-acceptor separation and angle. Results in Figure 14a for $\tau_{forward}$ show that the hydrogen bonds developed in water–THAL mixtures have very different lifespans. THAL-THAL hydrogen bonds, independently of the considered hydroxyl group involved in the interaction, have $\tau_{forward}$ an order of magnitude larger than any other hydrogen bond in the full composition range. The lifespan for all the considered hydrogen bonds increases with increasing THAL content in a non-linear way, which can be related with the development of larger THAL clusters, which decreases molecular mobility; Figure 6. It should be remarked that water-water hydrogen bonds have the lower lifespans, but they are also quickly reformed, Figure 14b. THAL-THAL hydrogen bonds have large lifespans, but the reformation time is even larger than the $\tau_{forward}$; thus, once THAL-THAL hydrogen bonds are broken, it is difficult to find a new suitable position to develop a new hydrogen bond, which is again related with the giant clusters formed for THAL rich mixtures, which decrease molecular mobility and also molecular reorientation forming hydrogen bonding reformation. THAL-water hydrogen bonds show similar properties considering THAL as donor or as acceptor, with lifespans in between the THAL-THAL and water-water interactions. The non-linear increase of kinetic properties for THAL-water interactions is again related with the THAL clustering considering that for THAL rich mixtures, once the large THAL clusters are formed, most of the water–THAL interactions will be developed with THAL molecules in the external shells of the THAL clusters. Regarding the temperature effect (Figure 15), the effect of *T*_g_ is inferred with extremely large lifespans for all the considered interactions below *T*_g_, which increase upon heating above *T*_g_. Likewise, reformation times are very large for low temperatures (it was not possible to measure it for 100 K).

**Figure 16:**
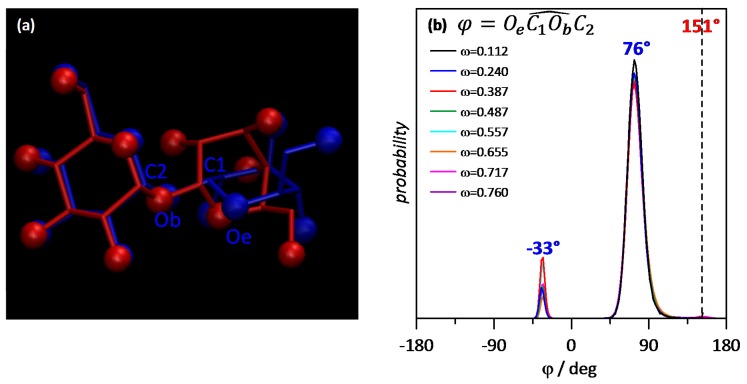
(**a**) Comparison of (red) structure of THAL molecule optimized (DFT) in gas phase and (blue) average structure of THAL molecule in ω THAL + (1-ω) water mixtures (ω = 0.112) at 310 K. Oxygen atoms are showed as van der Waals spheres; hydrogen atoms are omitted for the sake of visibility. (**b**) Probability distribution plots of the reported dihedral angle in ω THAL + (1-ω) water mixtures at 310 K. In panel b, dashed line shows the value of the dihedral in gas phase, and the peaks correspond to ω THAL + (1-ω) water mixtures.

The trend of molecules to self-aggregate forming large clusters should lead to changes in the properties of THAL molecule structure. Results in Figure 16a compare the average structure of THAL in water-THAL mixtures (ω = 0.112, *T* = 110 K) with the structure obtained in gas phase from DFT results. This comparison shows that THAL molecule in water-THAL mixtures is very different to that in gas phase, whereas in gas phase, both hexose rings are close to coplanar (151° for the dihedral angle around the glycoside oxygen, Figure 16a,b); in the case of water-THAL, one of the hexose rings suffers a rotation remaining almost perpendicular to the other ring; Figure 16a. Additionally, MD simulations of isolated THAL molecules (i.e., gas phase MD simulations) were carried out leading to 140 ± 9.5° for the dihedral angle around the glycoside oxygen, which are similar to those from DFT results and points to rotation of the THAL rings as produced by water presence. This rotation upon mixing with water is quantified in Figure 16b; the distribution of the dihedral angle is almost independent of the THAL content; thus, hexose rotation is inferred even for low THAL content. The reason of this THAL configuration in water mixtures could be the formation of a conformer, which allows more efficient THAL packing for the formation of THAL large clusters and allowing THAL-THAL hydrogen bonding in a more efficient way, thus fulfilling the THAL trend to self-associate.

**Figure 17:**
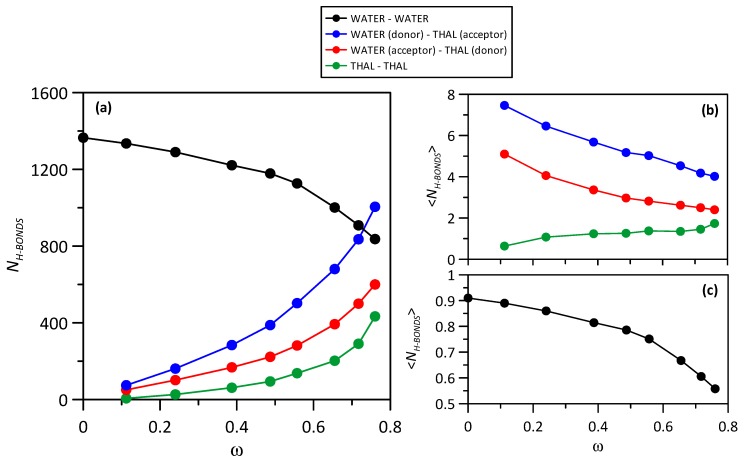
(**a**) Total number of hydrogen bonds in the simulation cell, *N_H-BONDS_*, and (**b**,**c**) number of hydrogen bonds per molecule, < *N_H-BONDS_* >, for the reported pairs in ω THAL + (1-ω) water mixtures (ω = 0.112) at 310 K. In panel b, results are reported per THAL molecule except for water-water hydrogen bonds.

The extension of hydrogen bonding in water-THAL mixtures is quantified in Figure 17, using the same hydrogen bonding criteria as for the kinetic properties in Figure 14 and Figure 15. Results in Figure 17a show the evolution of total number of hydrogen bonds in the simulation cell for the different interacting pairs. The non-linear evolution of hydrogen bonds with mixture composition stands on the large non-ideal behaviour of the water-THAL mixtures. Likewise, the strong affinity of water molecules for THAL molecules is also quantified as well as the extension of THAL-THAL hydrogen bonding. The average number of hydrogen bonds per molecule are reported in Figure 17b,c. Fort THAL-THAL interactions, a low number of hydrogen bonds per THAL molecule is inferred, starting from one hydrogen bond per molecule at low THAL content and evolving to two with increasing THAL content. Therefore, the clustering of THAL molecules is not characterized by a large extension of THAL-THAL hydrogen bonds; each THAL molecule is connected to another THAL molecule by a small number of hydrogen bonds, which are forming a network of hydrogen bonds leading to the large THAL clusters. The THAL molecules are also largely hydrogen bonded to surrounding water molecules both acting as donor and acceptor; Figure 17b. The increasing THAL concentration decreases water-THAL hydrogen molecules; as the THAL cluster size increases, the THAL hydroxyl sites are partially hidden into the cluster structure, thus hindering hydrogen bonding with water molecules. It is confirmed that for rich THAL mixtures, THAL-THAL interactions are just two per molecule whereas the number of water-THAL interactions has decreased by half when compared with water rich mixtures. In the case of water-water hydrogen bonding, it is decreased by half with increasing THAL content in comparison with water rich mixtures; water molecules tend to be hydrogen bonded with THAL molecules, which hinders water-water interactions with increasing THAL content. Therefore, the considered mixtures are characterized by hydrogen bonding networks involving THAL molecules, leading to THAL cluster, with water molecules hydrogen bonded to the remaining free THAL hydroxyl sites, developing water-THAL hydrogen bonds mainly with THAL molecules in the external regions of cluster, thus hindering water-water interactions.

## 3. Methods

Density functional theory (DFT) calculations were carried out to analyse THAL-water hydrogen bonding using around central THAL molecules considering small 1 THAL + *i* water molecular clusters (*i* = 1 to 3). These simulations allow accurate characterization of hydrogen bonding between the considered molecules in terms of interaction energy and the topology of the interacting sites as a function of the possible donor and acceptor sites. DFT modelling was carried out using ORCA program [22] at B3LYP [23,24,25] functional and 6-311++G(d, p) basis set, with dispersion correction from semiempirical Grimme’s method (DFT-D3) [26]. Interaction energies, Δ*E*, for the studied 1 THAL + *i* water clusters were calculated as the difference of the energy for the cluster and the sum of the energies of the corresponding monomers, at the same theoretical level, with Basis Set Superposition Error (BSSE) corrected through counterpoise method [27].

Classical MD simulations were carried out with MDynaMix v.5.2 [28] program. The forcefield parameterization for THAL is reported in Table S2 (Supplementary Material); charges and internal terms (bonds, angles and dihedrals) were obtained from Merck Molecular ForceField (MMFF) [29] whereas Lennard–Jones terms were obtained from CHARMM22 [30] forcefield as included in SwissParam database [31]. Water was described according to SPW-flexible water model [32]. The systems used for MD simulations, as described in Table S1 (Supplementary Material) were prepared considering a fixed number of water molecules (1500) and variable number of THAL molecules covering the 0 to 0.760 THAL mass fraction (ω). Initial simulation boxes were built with Packmol [33] program. Periodic boundary conditions were used for all MD simulations. Simulations were done in the NPT ensemble at 1 bar and temperatures in the 100 to 400 K range (100, 150, 180, 200, 230, 270, 310, 340, 370 and 400 K). Equilibration steps (2 ns) were followed by 20 ns long production runs. The equations of motion were treated using the Tuckerman–Berne double time step algorithm [34] (1 and 0.1 fs for long- and short-time steps). Coulombic terms were handled with the Ewald method [35], the number of terms in the reciprocal part was selected considering that the reciprocal series were cut when expression in the *exp* of the reciprocal part exceeds 9. For Lennard–Jones interactions, 15 Å cut-off was considered, and cross-terms were calculated with Lorentz–Berthelot mixing rules for all interacting pairs. No scaling factor was used for 1-4 Lennard–Jones interactions. This approach allowed covering both different Trehalose weight percentage (0% to 100%) and the temperature range. The objective of this approach was to mimic the drying process and the structural changes upon water content reduction. Although Lammert et al. [36] reported THAL solubility in water being 46.6 g THAL per 100 g solution, studies in the full composition range are required to study dehydration process. In the literature, previous MD studies have carried out MD simulations for THAL concentration well above 46.6 wt % [19,37]. Olgenblum et al. [29] used an analogous procedure to the one reported in this work to study Trehalose–water mixtures in the 0 to 100 wt%.

A relevant property for the study of THAL-water mixtures is the glass transition temperature, *T_g_*, as a function of solution concentration. The MD simulation of *T_g_*, is a quite challenging task [38], and it may be largely dependent on the considered cooling rate. Therefore, to avoid this artefact, the considered computational procedure, analogous to Olgenblum et al. [29], does not consider a particular cooling rate, generally employed in previous MD studies, but simulations for each of ten different temperatures (ranging from 100 K–470 K) and for each of the trehalose-water mixture composition. This procedure allowed enough equilibration for each temperature then followed by simulations at lower temperatures. Subsequently, the evolution of the predicted density for the different trehalose-water mixture at temperature range between 100–400 K, allows to infer *T_g_* values for each solution concentration. The *T_g_* values were determined from the temperature evolution of density for each mixture composition, although the most common approaches for the determination of *T_g_* stand on the evolution of properties such as heat capacity, thermal expansion coefficient, diffusion coefficient or specific volume [29]; the use of density has also been considered in the literature [39].

## 4. Conclusions

The properties of water + trehalose mixtures were studied as a function of trehalose content and temperature using computational chemistry tools. The reported results allowed the prediction of glass transition temperature, thus confirming completely different behaviour above and below it. The structure of the considered mixtures is characterized by the large trend of trehalose molecules paired with the great affinity of water molecules to be hydrogen bonded with trehalose hydroxyl sites. Trehalose’s trend to self-cluster leads to heterogeneous liquid mixtures with trehalose interacting with like molecules through a reduced number of hydrogen bonds but leading to networks of hydrogen bonds. The trehalose structure in water mixtures is characterized by the non-planarity of the two hexose rings in the molecule, which allow efficient packing in clusters with a small number of hydrogen bonds. Water molecules are largely hydrogen bonded to the available free hydroxyl sites in trehalose clusters. The trehalose–trehalose hydrogen bonds show large lifespans but also large reformation times, i.e., these hydrogen bonded networks show slow dynamics. Therefore, the effect of trehalose on the considered mixtures, and thus on the behaviour of trehalose for dry preservation purposes, stands on the formation on the trehalose clusters, even for low trehalose concentration, accompanied by very efficient water–trehalose interactions, which weakens the extension of water-water hydrogen bonding. The properties of trehalose-water mixtures, especially the clustering and hydrogen bonding extension, may be fine-tuned by the selection of proper composition and temperature conditions, which is of large relevance for practical purposes.

## Supplementary Materials

The Supplementary Materials are available online. This section includes: Table S1 (description of systems used for molecular dynamics simulations); Table S2 (force field parameterizations used along this work); Figure S1 (comparison of trehalose structure).
