# Supplementary material for: A Theoretical Study on Trehalose + Water Mixtures for Dry Preservation Purposes
Source: Molecules. 2020 Mar 21;25(6):1435. doi: 10.3390/molecules25061435 (PMC7145318; doi:10.3390/molecules25061435)
Supplement: Supplementary file 1 [file molecules-25-01435-s001.pdf]

Electronic Supplementary Information

A Theoretical Study on Trehalose + Water Mixtures for Dry Preservation Purposes

Amit Kumar,[a] Alberto Cincotti,[b] Santiago Aparicio*[c]

[a] Department of Electrical and Electronic Engineering, University of Cagliari, 09123 Cagliari, Italy

[b] Department of Mechanical, Chemical and Materials Engineering, University of Cagliari, 09123 Cagliari, Italy

[c] Department of Chemistry, University of Burgos, 09001 Burgos, Spain

*Corresponding author: sapar@ubu.es

Table S1. Compositions of THAL–water mixtures subjected to MD simulations. ω stands for THAL mass fraction. All the systems were subjected to NPT simulations at 100, 150, 180, 200, 230, 270, 310, 340, 370 and 400 K

systems	N_{THAL}	N_{water}	ω
1	250	1500	0.760
2	200	1500	0.717
3	150	1500	0.655
4	100	1500	0.557
5	75	1500	0.487
6	50	1500	0.387
7	25	1500	0.240
8	10	1500	0.112
9	0	1500	0
10	150	0	1

Table S2. Forcefield parameterization for compounds studied in this work.

The general form of the applied force field is:

$$E = \sum_{bonds} k_r (r - r_{eq})^2 + \sum_{angles} k_\theta (\theta - \theta_{eq})^2 + E_{tor}$$

$$+ \sum_i \sum_j \left\{ 4\varepsilon_{ij} \left[\left(\frac{\sigma_{ij}}{r_{ij}} \right)^{12} - \left(\frac{\sigma_{ij}}{r_{ij}} \right)^{6} \right] + \frac{q_i q_j e^2}{4\pi\varepsilon_0 r_{ij}} \right\}$$

Dihedrals (E_{tor}) were described according to:

$$E_{tor} = \sum_{torsions} k_\phi (1 + \cos(m\phi - \delta))$$

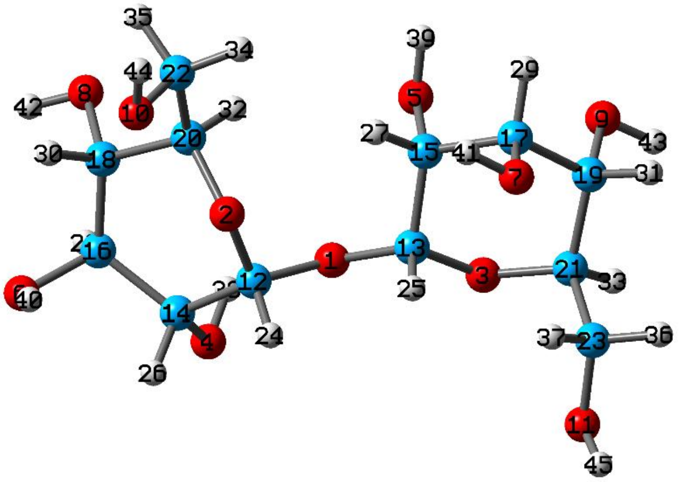

[THAL]

atom	q	σ_{ii} / Å	ε_{ii} / kJ mol^{-1}	#
O	-0.5600	3.153780	0.636386	1
O	-0.5600	3.153780	0.636386	2
O	-0.5600	3.153780	0.636386	3
O	-0.6800	3.153780	0.636386	4
O	-0.6800	3.153780	0.636386	5
O	-0.6800	3.153780	0.636386	6
O	-0.6800	3.153780	0.636386	7
O	-0.6800	3.153780	0.636386	8
O	-0.6800	3.153780	0.636386	9
O	-0.6800	3.153780	0.636386	10
O	-0.6800	3.153780	0.636386	11
C	0.5600	3.875410	0.230120	12
C	0.5600	3.875410	0.230120	13
C	0.2800	3.875410	0.230120	14
C	0.2800	3.875410	0.230120	15
C	0.2800	3.875410	0.230120	16
C	0.2800	3.875410	0.230120	17
C	0.2800	3.875410	0.230120	18
C	0.2800	3.875410	0.230120	19

C	0.2800	3.875410	0.230120	20
C	0.2800	3.875410	0.230120	21
C	0.2800	3.875410	0.230120	22
C	0.2800	3.875410	0.230120	23
H	0.0000	2.351970	0.092048	24
H	0.0000	2.351970	0.092048	25
H	0.0000	2.351970	0.092048	26
H	0.0000	2.351970	0.092048	27
H	0.0000	2.351970	0.092048	28
H	0.0000	2.351970	0.092048	29
H	0.0000	2.351970	0.092048	30
H	0.0000	2.351970	0.092048	31
H	0.0000	2.351970	0.092048	32
H	0.0000	2.351970	0.092048	33
H	0.0000	2.351970	0.092048	34
H	0.0000	2.351970	0.092048	35
H	0.0000	2.351970	0.092048	36
H	0.0000	2.351970	0.092048	37
H	0.4000	0.400010	0.192464	38
H	0.4000	0.400010	0.192464	39
H	0.4000	0.400010	0.192464	40
H	0.4000	0.400010	0.192464	41
H	0.4000	0.400010	0.192464	42
H	0.4000	0.400010	0.192464	43
H	0.4000	0.400010	0.192464	44
H	0.4000	0.400010	0.192464	45

Bonds

**Atom Numbers**		r_{eq} /Å	k_r / kJ mol^{-1} Å^{-2}
1	12	1.4180	1519.6875
1	13	1.4180	1519.6875
2	12	1.4180	1519.6875
2	20	1.4180	1519.6875
3	13	1.4180	1519.6875
3	21	1.4180	1519.6875
4	14	1.4180	1519.6875
4	38	0.9720	2346.8265
5	15	1.4180	1519.6875
5	39	0.9720	2346.8265
6	16	1.4180	1519.6875
6	40	0.9720	2346.8265
7	17	1.4180	1519.6875
7	41	0.9720	2346.8265
8	18	1.4180	1519.6875
8	42	0.9720	2346.8265
9	19	1.4180	1519.6875
9	43	0.9720	2346.8265
10	22	1.4180	1519.6875
10	44	0.9720	2346.8265
11	23	1.4180	1519.6875
11	45	0.9720	2346.8265
12	14	1.5080	1282.1115
12	24	1.0930	1435.0745
13	15	1.5080	1282.1115

13	25	1.0930	1435.0745
14	16	1.5080	1282.1115
14	26	1.0930	1435.0745
15	17	1.5080	1282.1115
15	27	1.0930	1435.0745
16	18	1.5080	1282.1115
16	28	1.0930	1435.0745
17	19	1.5080	1282.1115
17	29	1.0930	1435.0745
18	20	1.5080	1282.1115
18	30	1.0930	1435.0745
19	21	1.5080	1282.1115
19	31	1.0930	1435.0745
20	22	1.5080	1282.1115
20	32	1.0930	1435.0745
21	23	1.5080	1282.1115
21	33	1.0930	1435.0745
22	34	1.0930	1435.0745
22	35	1.0930	1435.0745
23	36	1.0930	1435.0745
23	37	1.0930	1435.0745

Angles

**Atom Numbers**			**θ_{eq} / deg**	**k_ϑ / kJ mol^{-1} rad^{-2}**
12	1	13	106.926	720.840
12	2	20	106.926	720.840
13	3	21	106.926	720.840
14	4	38	106.503	477.550
15	5	39	106.503	477.550
16	6	40	106.503	477.550
17	7	41	106.503	477.550
18	8	42	106.503	477.550
19	9	43	106.503	477.550
22	10	44	106.503	477.550
23	11	45	106.503	477.550
1	12	2	111.368	696.150
1	12	14	108.133	597.390
1	12	24	108.577	470.320
2	12	14	108.133	597.390
2	12	24	108.577	470.320
14	12	24	110.549	383.000
1	13	3	111.368	696.150
1	13	15	108.133	597.390
1	13	25	108.577	470.320
3	13	15	108.133	597.390
3	13	25	108.577	470.320
15	13	25	110.549	383.000
4	14	12	108.133	597.390
4	14	16	108.133	597.390
4	14	26	108.577	470.320
12	14	16	109.608	512.480
12	14	26	110.549	383.000
16	14	26	110.549	383.000

5	15	13	108.133	597.390
5	15	17	108.133	597.390
5	15	27	108.577	470.320
13	15	17	109.608	512.480
13	15	27	110.549	383.000
17	15	27	110.549	383.000
6	16	14	108.133	597.390
6	16	18	108.133	597.390
6	16	28	108.577	470.320
14	16	18	109.608	512.480
14	16	28	110.549	383.000
18	16	28	110.549	383.000
7	17	15	108.133	597.390
7	17	19	108.133	597.390
7	17	29	108.577	470.320
15	17	19	109.608	512.480
15	17	29	110.549	383.000
19	17	29	110.549	383.000
8	18	16	108.133	597.390
8	18	20	108.133	597.390
8	18	30	108.577	470.320
16	18	20	109.608	512.480
16	18	30	110.549	383.000
20	18	30	110.549	383.000
9	19	17	108.133	597.390
9	19	21	108.133	597.390
9	19	31	108.577	470.320
17	19	21	109.608	512.480
17	19	31	110.549	383.000
21	19	31	110.549	383.000
2	20	18	108.133	597.390
2	20	22	108.133	597.390
2	20	32	108.577	470.320
18	20	22	109.608	512.480
18	20	32	110.549	383.000
22	20	32	110.549	383.000
3	21	19	108.133	597.390
3	21	23	108.133	597.390
3	21	33	108.577	470.320
19	21	23	109.608	512.480
19	21	33	110.549	383.000
23	21	33	110.549	383.000
10	22	20	108.133	597.390
10	22	34	108.577	470.320
10	22	35	108.577	470.320
20	22	34	110.549	383.000
20	22	35	110.549	383.000
34	22	35	108.836	310.740
11	23	21	108.133	597.390
11	23	36	108.577	470.320
11	23	37	108.577	470.320
21	23	36	110.549	383.000
21	23	37	110.549	383.000
36	23	37	108.836	310.740

Dihedrals

Atom Numbers				δ / deg	k_φ / kJ mol^{-1}	m
1	12	2	20	0	0.4812	1
1	12	2	20	180	-1.4853	2
1	12	2	20	0	1.5104	3
1	12	14	4	0	0.8535	1
1	12	14	4	180	2.9246	2
1	12	14	4	0	2.0083	3
1	12	14	16	0	-1.4393	1
1	12	14	16	180	3.6777	2
1	12	14	16	0	0.9958	3
1	12	14	26	0	-1.3682	1
1	12	14	26	180	2.2426	2
1	12	14	26	0	0.5858	3
1	13	3	21	0	0.4812	1
1	13	3	21	180	-1.4853	2
1	13	3	21	0	1.5104	3
1	13	15	5	0	0.8535	1
1	13	15	5	180	2.9246	2
1	13	15	5	0	2.0083	3
1	13	15	17	0	-1.4393	1
1	13	15	17	180	3.6777	2
1	13	15	17	0	0.9958	3
1	13	15	27	0	-1.3682	1
1	13	15	27	180	2.2426	2
1	13	15	27	0	0.5858	3
2	12	1	13	0	0.4812	1
2	12	1	13	180	-1.4853	2
2	12	1	13	0	1.5104	3
2	12	14	4	0	0.8535	1
2	12	14	4	180	2.9246	2
2	12	14	4	0	2.0083	3
2	12	14	16	0	-1.4393	1
2	12	14	16	180	3.6777	2
2	12	14	16	0	0.9958	3
2	12	14	26	0	-1.3682	1
2	12	14	26	180	2.2426	2
2	12	14	26	0	0.5858	3
2	20	18	8	0	0.8535	1
2	20	18	8	180	2.9246	2
2	20	18	8	0	2.0083	3
2	20	18	16	0	-1.4393	1
2	20	18	16	180	3.6777	2
2	20	18	16	0	0.9958	3
2	20	18	30	0	-1.3682	1
2	20	18	30	180	2.2426	2
2	20	18	30	0	0.5858	3
2	20	22	10	0	0.8535	1
2	20	22	10	180	2.9246	2
2	20	22	10	0	2.0083	3
2	20	22	34	0	-1.3682	1
2	20	22	34	180	2.2426	2
2	20	22	34	0	0.5858	3

2	20	22	35	0	-1.3682	1
2	20	22	35	180	2.2426	2
2	20	22	35	0	0.5858	3
3	13	1	12	0	0.4812	1
3	13	1	12	180	-1.4853	2
3	13	1	12	0	1.5104	3
3	13	15	5	0	0.8535	1
3	13	15	5	180	2.9246	2
3	13	15	5	0	2.0083	3
3	13	15	17	0	-1.4393	1
3	13	15	17	180	3.6777	2
3	13	15	17	0	0.9958	3
3	13	15	27	0	-1.3682	1
3	13	15	27	180	2.2426	2
3	13	15	27	0	0.5858	3
3	21	19	9	0	0.8535	1
3	21	19	9	180	2.9246	2
3	21	19	9	0	2.0083	3
3	21	19	17	0	-1.4393	1
3	21	19	17	180	3.6777	2
3	21	19	17	0	0.9958	3
3	21	19	31	0	-1.3682	1
3	21	19	31	180	2.2426	2
3	21	19	31	0	0.5858	3
3	21	23	11	0	0.8535	1
3	21	23	11	180	2.9246	2
3	21	23	11	0	2.0083	3
3	21	23	36	0	-1.3682	1
3	21	23	36	180	2.2426	2
3	21	23	36	0	0.5858	3
3	21	23	37	0	-1.3682	1
3	21	23	37	180	2.2426	2
3	21	23	37	0	0.5858	3
4	14	12	24	0	-1.3682	1
4	14	12	24	180	2.2426	2
4	14	12	24	0	0.5858	3
4	14	16	6	0	0.8535	1
4	14	16	6	180	2.9246	2
4	14	16	6	0	2.0083	3
4	14	16	18	0	-1.4393	1
4	14	16	18	180	3.6777	2
4	14	16	18	0	0.9958	3
4	14	16	28	0	-1.3682	1
4	14	16	28	180	2.2426	2
4	14	16	28	0	0.5858	3
5	15	13	25	0	-1.3682	1
5	15	13	25	180	2.2426	2
5	15	13	25	0	0.5858	3
5	15	17	7	0	0.8535	1
5	15	17	7	180	2.9246	2
5	15	17	7	0	2.0083	3
5	15	17	19	0	-1.4393	1
5	15	17	19	180	3.6777	2
5	15	17	19	0	0.9958	3

5	15	17	29	0	-1.3682	1
5	15	17	29	180	2.2426	2
5	15	17	29	0	0.5858	3
6	16	14	12	0	-1.4393	1
6	16	14	12	180	3.6777	2
6	16	14	12	0	0.9958	3
6	16	14	26	0	-1.3682	1
6	16	14	26	180	2.2426	2
6	16	14	26	0	0.5858	3
6	16	18	8	0	0.8535	1
6	16	18	8	180	2.9246	2
6	16	18	8	0	2.0083	3
6	16	18	20	0	-1.4393	1
6	16	18	20	180	3.6777	2
6	16	18	20	0	0.9958	3
6	16	18	30	0	-1.3682	1
6	16	18	30	180	2.2426	2
6	16	18	30	0	0.5858	3
7	17	15	13	0	-1.4393	1
7	17	15	13	180	3.6777	2
7	17	15	13	0	0.9958	3
7	17	15	27	0	-1.3682	1
7	17	15	27	180	2.2426	2
7	17	15	27	0	0.5858	3
7	17	19	9	0	0.8535	1
7	17	19	9	180	2.9246	2
7	17	19	9	0	2.0083	3
7	17	19	21	0	-1.4393	1
7	17	19	21	180	3.6777	2
7	17	19	21	0	0.9958	3
7	17	19	31	0	-1.3682	1
7	17	19	31	180	2.2426	2
7	17	19	31	0	0.5858	3
8	18	16	14	0	-1.4393	1
8	18	16	14	180	3.6777	2
8	18	16	14	0	0.9958	3
8	18	16	28	0	-1.3682	1
8	18	16	28	180	2.2426	2
8	18	16	28	0	0.5858	3
8	18	20	22	0	-1.4393	1
8	18	20	22	180	3.6777	2
8	18	20	22	0	0.9958	3
8	18	20	32	0	-1.3682	1
8	18	20	32	180	2.2426	2
8	18	20	32	0	0.5858	3
9	19	17	15	0	-1.4393	1
9	19	17	15	180	3.6777	2
9	19	17	15	0	0.9958	3
9	19	17	29	0	-1.3682	1
9	19	17	29	180	2.2426	2
9	19	17	29	0	0.5858	3
9	19	21	23	0	-1.4393	1
9	19	21	23	180	3.6777	2
9	19	21	23	0	0.9958	3

9	19	21	33	0	-1.3682	1
9	19	21	33	180	2.2426	2
9	19	21	33	0	0.5858	3
10	22	20	18	0	-1.4393	1
10	22	20	18	180	3.6777	2
10	22	20	18	0	0.9958	3
10	22	20	32	0	-1.3682	1
10	22	20	32	180	2.2426	2
10	22	20	32	0	0.5858	3
11	23	21	19	0	-1.4393	1
11	23	21	19	180	3.6777	2
11	23	21	19	0	0.9958	3
11	23	21	33	0	-1.3682	1
11	23	21	33	180	2.2426	2
11	23	21	33	0	0.5858	3
12	1	13	15	0	-1.4267	1
12	1	13	15	180	1.5816	2
12	1	13	15	0	1.5816	3
12	1	13	25	0	1.1924	1
12	1	13	25	180	0.6694	2
12	1	13	25	0	1.1924	3
12	2	20	18	0	-1.4267	1
12	2	20	18	180	1.5816	2
12	2	20	18	0	1.5816	3
12	2	20	22	0	-1.4267	1
12	2	20	22	180	1.5816	2
12	2	20	22	0	1.5816	3
12	2	20	32	0	1.1924	1
12	2	20	32	180	0.6694	2
12	2	20	32	0	1.1924	3
12	14	4	38	180	0.5648	2
12	14	4	38	0	0.4937	3
12	14	16	18	0	0.2134	1
12	14	16	18	180	1.4267	2
12	14	16	18	0	0.6945	3
12	14	16	28	0	1.3389	1
12	14	16	28	180	-1.3180	2
12	14	16	28	0	0.5523	3
13	1	12	14	0	-1.4267	1
13	1	12	14	180	1.5816	2
13	1	12	14	0	1.5816	3
13	1	12	24	0	1.1924	1
13	1	12	24	180	0.6694	2
13	1	12	24	0	1.1924	3
13	3	21	19	0	-1.4267	1
13	3	21	19	180	1.5816	2
13	3	21	19	0	1.5816	3
13	3	21	23	0	-1.4267	1
13	3	21	23	180	1.5816	2
13	3	21	23	0	1.5816	3
13	3	21	33	0	1.1924	1
13	3	21	33	180	0.6694	2
13	3	21	33	0	1.1924	3
13	15	5	39	180	0.5648	2

13	15	5	39	0	0.4937	3
13	15	17	19	0	0.2134	1
13	15	17	19	180	1.4267	2
13	15	17	19	0	0.6945	3
13	15	17	29	0	1.3389	1
13	15	17	29	180	-1.3180	2
13	15	17	29	0	0.5523	3
14	12	2	20	0	-1.4267	1
14	12	2	20	180	1.5816	2
14	12	2	20	0	1.5816	3
14	16	6	40	180	0.5648	2
14	16	6	40	0	0.4937	3
14	16	18	20	0	0.2134	1
14	16	18	20	180	1.4267	2
14	16	18	20	0	0.6945	3
14	16	18	30	0	1.3389	1
14	16	18	30	180	-1.3180	2
14	16	18	30	0	0.5523	3
15	13	3	21	0	-1.4267	1
15	13	3	21	180	1.5816	2
15	13	3	21	0	1.5816	3
15	17	7	41	180	0.5648	2
15	17	7	41	0	0.4937	3
15	17	19	21	0	0.2134	1
15	17	19	21	180	1.4267	2
15	17	19	21	0	0.6945	3
15	17	19	31	0	1.3389	1
15	17	19	31	180	-1.3180	2
15	17	19	31	0	0.5523	3
16	14	4	38	180	0.5648	2
16	14	4	38	0	0.4937	3
16	14	12	24	0	1.3389	1
16	14	12	24	180	-1.3180	2
16	14	12	24	0	0.5523	3
16	18	8	42	180	0.5648	2
16	18	8	42	0	0.4937	3
16	18	20	22	0	0.2134	1
16	18	20	22	180	1.4267	2
16	18	20	22	0	0.6945	3
16	18	20	32	0	1.3389	1
16	18	20	32	180	-1.3180	2
16	18	20	32	0	0.5523	3
17	15	5	39	180	0.5648	2
17	15	5	39	0	0.4937	3
17	15	13	25	0	1.3389	1
17	15	13	25	180	-1.3180	2
17	15	13	25	0	0.5523	3
17	19	9	43	180	0.5648	2
17	19	9	43	0	0.4937	3
17	19	21	23	0	0.2134	1
17	19	21	23	180	1.4267	2
17	19	21	23	0	0.6945	3
17	19	21	33	0	1.3389	1
17	19	21	33	180	-1.3180	2

17	19	21	33	0	0.5523	3
18	16	6	40	180	0.5648	2
18	16	6	40	0	0.4937	3
18	16	14	26	0	1.3389	1
18	16	14	26	180	-1.3180	2
18	16	14	26	0	0.5523	3
18	20	22	34	0	1.3389	1
18	20	22	34	180	-1.3180	2
18	20	22	34	0	0.5523	3
18	20	22	35	0	1.3389	1
18	20	22	35	180	-1.3180	2
18	20	22	35	0	0.5523	3
19	17	7	41	180	0.5648	2
19	17	7	41	0	0.4937	3
19	17	15	27	0	1.3389	1
19	17	15	27	180	-1.3180	2
19	17	15	27	0	0.5523	3
19	21	23	36	0	1.3389	1
19	21	23	36	180	-1.3180	2
19	21	23	36	0	0.5523	3
19	21	23	37	0	1.3389	1
19	21	23	37	180	-1.3180	2
19	21	23	37	0	0.5523	3
20	2	12	24	0	1.1924	1
20	2	12	24	180	0.6694	2
20	2	12	24	0	1.1924	3
20	18	8	42	180	0.5648	2
20	18	8	42	0	0.4937	3
20	18	16	28	0	1.3389	1
20	18	16	28	180	-1.3180	2
20	18	16	28	0	0.5523	3
20	22	10	44	180	0.5648	2
20	22	10	44	0	0.4937	3
21	3	13	25	0	1.1924	1
21	3	13	25	180	0.6694	2
21	3	13	25	0	1.1924	3
21	19	9	43	180	0.5648	2
21	19	9	43	0	0.4937	3
21	19	17	29	0	1.3389	1
21	19	17	29	180	-1.3180	2
21	19	17	29	0	0.5523	3
21	23	11	45	180	0.5648	2
21	23	11	45	0	0.4937	3
22	20	18	30	0	1.3389	1
22	20	18	30	180	-1.3180	2
22	20	18	30	0	0.5523	3
23	21	19	31	0	1.3389	1
23	21	19	31	180	-1.3180	2
23	21	19	31	0	0.5523	3
24	12	14	26	0	0.5941	1
24	12	14	26	180	-2.8995	2
24	12	14	26	0	0.6569	3
25	13	15	27	0	0.5941	1
25	13	15	27	180	-2.8995	2

25	13	15	27	0	0.6569	3
26	14	4	38	0	1.2468	1
26	14	4	38	180	-0.5774	2
26	14	4	38	0	0.7238	3
26	14	16	28	0	0.5941	1
26	14	16	28	180	-2.8995	2
26	14	16	28	0	0.6569	3
27	15	5	39	0	1.2468	1
27	15	5	39	180	-0.5774	2
27	15	5	39	0	0.7238	3
27	15	17	29	0	0.5941	1
27	15	17	29	180	-2.8995	2
27	15	17	29	0	0.6569	3
28	16	6	40	0	1.2468	1
28	16	6	40	180	-0.5774	2
28	16	6	40	0	0.7238	3
28	16	18	30	0	0.5941	1
28	16	18	30	180	-2.8995	2
28	16	18	30	0	0.6569	3
29	17	7	41	0	1.2468	1
29	17	7	41	180	-0.5774	2
29	17	7	41	0	0.7238	3
29	17	19	31	0	0.5941	1
29	17	19	31	180	-2.8995	2
29	17	19	31	0	0.6569	3
30	18	8	42	0	1.2468	1
30	18	8	42	180	-0.5774	2
30	18	8	42	0	0.7238	3
30	18	20	32	0	0.5941	1
30	18	20	32	180	-2.8995	2
30	18	20	32	0	0.6569	3
31	19	9	43	0	1.2468	1
31	19	9	43	180	-0.5774	2
31	19	9	43	0	0.7238	3
31	19	21	33	0	0.5941	1
31	19	21	33	180	-2.8995	2
31	19	21	33	0	0.6569	3
32	20	22	34	0	0.5941	1
32	20	22	34	180	-2.8995	2
32	20	22	34	0	0.6569	3
32	20	22	35	0	0.5941	1
32	20	22	35	180	-2.8995	2
32	20	22	35	0	0.6569	3
33	21	23	36	0	0.5941	1
33	21	23	36	180	-2.8995	2
33	21	23	36	0	0.6569	3
33	21	23	37	0	0.5941	1
33	21	23	37	180	-2.8995	2
33	21	23	37	0	0.6569	3
34	22	10	44	0	1.2468	1
34	22	10	44	180	-0.5774	2
34	22	10	44	0	0.7238	3
35	22	10	44	0	1.2468	1
35	22	10	44	180	-0.5774	2

35	22	10	44	0	0.7238	3
36	23	11	45	0	1.2468	1
36	23	11	45	180	-0.5774	2
36	23	11	45	0	0.7238	3
37	23	11	45	0	1.2468	1
37	23	11	45	180	-0.5774	2
37	23	11	45	0	0.7238	3

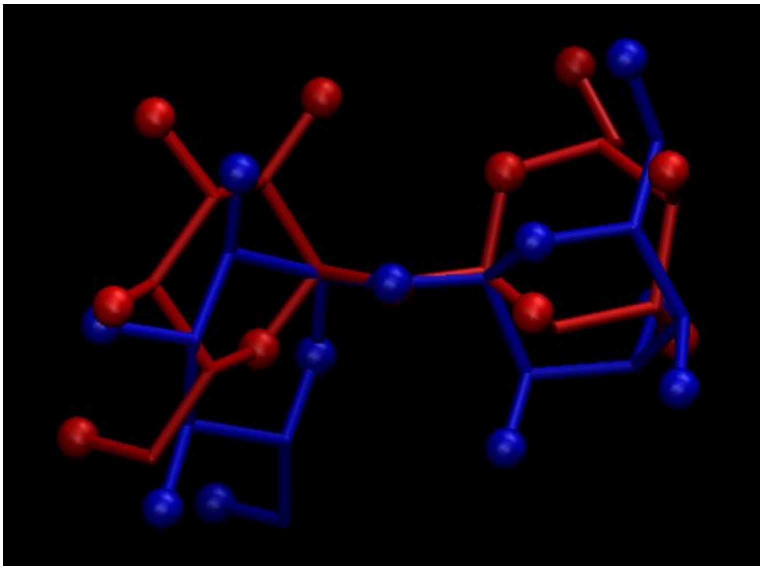

Figure S1. Comparison of (blue) THAL molecule DFT optimized in gas phase and (red) structure of THAL in 1 THAL : 1 water cluster, corresponding to the structure reported in Figure 2b. Hydrogen atoms and water molecule are omitted to improve visibility. Oxygen atoms are plotted as balls to improve visibility.
